# Temporal Clustering of Skin Sympathetic Nerve Activity Bursts in Acute Myocardial Infarction Patients

**DOI:** 10.3389/fnins.2021.720827

**Published:** 2021-11-30

**Authors:** Chun Liu, Chien-Hung Lee, Shien-Fong Lin, Wei-Chung Tsai

**Affiliations:** ^1^Department of Electrical and Computer Engineering, College of Electrical and Computer Engineering, National Chiao Tung University, Hsinchu, Taiwan; ^2^Department of Public Health, College of Health Sciences, Kaohsiung Medical University, Kaohsiung, Taiwan; ^3^Research Center for Environmental Medicine, Kaohsiung Medical University, Kaohsiung, Taiwan; ^4^Institute of Biomedical Engineering, College of Electrical and Computer Engineering, National Chiao Tung University, Hsinchu, Taiwan; ^5^Division of Cardiology, Department of Internal Medicine, Kaohsiung Medical University Hospital, Kaohsiung Medical University, Kaohsiung, Taiwan; ^6^Department of Internal Medicine, Faculty of Medicine, College of Medicine, Kaohsiung Medical University, Kaohsiung, Taiwan

**Keywords:** acute myocardial infarction, autonomic nervous system, clustering nerve activity, non-invasive measurement, neuECG

## Abstract

**Backgrounds:** Acute myocardial infarction (AMI) affects the autonomic nervous system (ANS) function. The aim of our study is to detect the particular patterns of ANS regulation in AMI. We hypothesize that altered ANS regulation in AMI patients causes synchronized neural discharge (clustering phenomenon) detected by non-invasive skin sympathetic nerve activity (SKNA).

**Methods:** Forty subjects, including 20 AMI patients and 20 non-AMI controls, participated in the study. The wide-band bioelectrical signals (neuECG) were continuously recorded on the body surface for 5 min. SKNA was signal processed to depict the envelope of SKNA (eSKNA). By labeling the clusters, the AMI subjects were separated into non-AMI, non-cluster appearing (AMI_NCA_), and cluster appearing (AMI_CA_) groups.

**Results:** The average eSKNA was significantly correlated with HRV low-frequency (LF) power (rho = −0.336) and high-frequency power (rho = −0.372). The cross-comparison results demonstrated that eSKNA is a valid surrogate marker to assess ANS in AMI patients. The frequency of cluster occurrence was 0.01–0.03 Hz and the amplitude was about 3 μV. The LF/HF ratio of AMI_CA_ (median: 1.877; Q1–Q3: 1.483–2.413) revealed significantly lower than AMI_NCA_ (median: 3.959; Q1–Q3: 1.840–6.562). The results suggest that the SKNA clustering is a unique temporal pattern of ANS synchronized discharge, which could indicate the lower sympathetic status (by HRV) in AMI patients.

**Conclusion:** This is the first study to identify SKNA clustering phenomenon in AMI patients. Such a synchronized nerve discharge pattern could be detected with non-invasive SKNA signals. SKNA temporal clustering could be a novel biomarker to classify ANS regulation ability in AMI patients.

**Clinical and Translational Significance:** SKNA is higher in AMI patients than in control and negatively correlates with parasympathetic parameters. SKNA clustering is associated with a lower LF/HF ratio that has been shown to correlate with sudden cardiac death in AMI. The lack of SKNA temporal clustering could indicate poor ANS regulation in AMI patients.

## Introduction

Acute myocardial infarction (AMI) is one of the leading causes of morbidity and mortality worldwide. AMI causes myocardial necrosis and affects cardiac function. The estimated global population with AMI was almost 16 million in 2015 and increased year after year (Disease et al., 2016). In the past decade, the autonomic regulation and the sudden cardiac death risk stratification of patients with AMI has become an important topic (Barron and Lesh, 1996). Autonomic regulation parameters such as baroreflex sensitivity, heart rate variability (HRV), and heart rate turbulence are significantly correlated with sudden cardiac death in AMI patients (La Rovere et al., 1998, 2001; Schmidt et al., 1999). Kasama et al. (2007) and Han et al. (2012) evaluate the variations of AMI patients in different aspects. It has been substantiated that AMI patients are accompanied by remodeling of sympathetic nervous system (SNS) and left ventricular structure. Therefore, the research on autonomic function assessment and prediction in autonomic nerve discharge in AMI patients is clinically relevant to predict patient outcome.

Sympathetic nerve activity is traditionally measured with microneurography, which utilizes a needle electrode inserted into a specific bundle of sympathetic nerves. Because of the specificity and exclusivity of electrode placement, the actual nerve activity can be observed and measured (Hagbarth and Vallbo, 1968). Microneurography has been applied to the measurement of invasive muscle sympathetic nerve activity (MSNA) in different subjects by Kingwell et al. (1994) and animal models by Jung et al. (2006). Ang and Marina (2020) suggested that MSNA is coupled with rhythmic fluctuations in blood pressure with physiological significance. In normal humans, MSNA is positively correlated with the low-frequency (LF) components of RR interval variability during sympathetic activation and inhibition (Pagani et al., 1997). MSNA is clustered in a series of bursts that are highly correlated with the spontaneous fluctuations of blood pressure (Mayer waves) under the tilt test (Furlan et al., 2000; Marchi et al., 2016). Recently, Chen’s group developed a novel non-invasive method based on the wide-band biosignal (neuECG), with which the small-scale skin sympathetic nerve activity (SKNA) signal can be extracted (Chen and Lin, 2015; Robinson et al., 2015; Everett et al., 2017; Kusayama et al., 2020b). However, the evidence of direct comparison between MSNA and SKNA is lacking. By microneurographic technique, Grassi et al. (1998) demonstrated the dissociation between MSNA and postganglionic sympathetic nerve activity in the skin area. MSNA is sensitive to blood pressure but not heart rate. On the other hand, SKNA is sensitive to heart rate and may be used in cardiac arrhythmias (Kabir et al., 2017; Kusayama et al., 2020a). Most of the analyses of SKNA focused on nerve discharge frequency, amplitude, and average nerve activity; almost none of the research investigated the temporal pattern or clustered series in SKNA.

Kusayama et al. (2019) demonstrated that large and sustained SKNA is associated with temporal clustering of atrial fibrillation, ventricular tachycardia, and ventricular fibrillation. In particular, our recent study has also detected SKNA discharge clustering in patients with paroxysmal atrial fibrillation (Liu et al., 2018). Since AMI affects the autonomic nervous system (ANS) regulation and is associated with arrhythmias, we hypothesize that a distinctive ANS regulation in AMI patients could cause synchronized neural discharge (clustering phenomenon) by the non-invasive SKNA. We aim to identify the pattern of SKNA clustering and its correlation with other ANS parameters in AMI patients.

## Materials and Methods

### Experimental Design

This trial (NCT03243448) was a cross-sectional, single-center, observational study approved by Institutional Review Board of Kaohsiung Medical University Hospital. Twenty AMI patients and twenty non-AMI subjects were enrolled as case and reference, respectively, in the study. All participants provided informed written consent before participating in the study. The SKNA is measured on the first morning after admission in the AMI patients and the morning during health check-up in the controls. The study participants rested in the supine position for at least 10 min before SKNA recording in the intensive care unit ward or ECG recording room in the health check-up department. The average temperature and moisture were approximately 23 ± 2°C and 50% ± 5% in the recording areas. The clinical characteristics of study subjects are shown in Table 1. The associations between SKNA, demographic, lifestyle, and clinical factors are shown in Supplementary Table 1.

**TABLE 1:** Comparison of clinical characteristics of study subjects.

**Characteristics**	**Non-AMI**		**AMI**		***p*-value**
	**No.**	**(%)**	**No.**	**(%)**	
**Total**	20		20		
**Gender**					
Male	20	(100)	20	(100)	
**Age (Mean ± SD), year**	51.9 ± 14.5		52.8 ± 13.5		0.444
<40	5	(25)	2	(10)	
40–59	8	(40)	11	(55)	
<60	7	(35)	7	(35)	
**Body Mass Index (kg/m^2^)**	23.6 ± 2.9		27.2 ± 4.2		0.338
<26	17	(85)	8	(40)	
>26	3	(15)	12	(60)	
**Mean blood pressure (mmHg)**	98.0 ± 9.4		101 ± 15.4		0.468
<95	7	(35)	12	(60)	
>95	13	(65)	8	(40)	
**Disease, yes**					
Hypertension	1	(5)	11	(55)	<0.001***
Diabetes mellitus	0	(0)	10	(50)	<0.001***
Dyslipidemia	5	(25)	16	(80)	<0.001***
**HFrEF, yes**		NA	5	(25)	NA
**Type of AMI**		NA			NA
STEMI			13	(65)	
Inferior wall STEMI			4	(31)	
Non-inferior wall STEMI			9	(69)	
NSTEMI		NA	7 (35)		NA

AMI, acute myocardial infarction; HFrEF, heart failure with reduced ejection fraction; NSTEMI, non-ST elevation myocardial infarction; STEMI, ST elevation myocardial infarction. ***Statistically significant (*p* < 0.001).

The 5-min baseline signal was recorded from the subjects using conventional ECG patch electrodes with a standard lead I placement. All data were acquired with a wide-band bioamplifier (2 kHz bandpass, Biomonitor ME6000, MEGA Electronics, Kuopio, Finland), which provided a high sampling rate (10 kHz) to meet the requirements of SKNA acquisition and processing (Chen and Lin, 2015; Kusayama et al., 2020b).

### Signal Processing of Skin Sympathetic Nerve Activity

**FIGURE 1:**
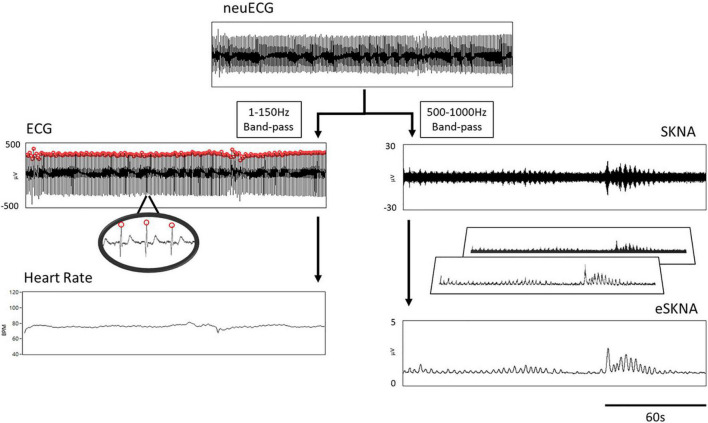
Flow chart of signal processing. The neuECG passed through a 1- to 150-Hz bandpass filter to show the ECG, which was processed by an R-wave peak detector to calculate heart rate (left path). The 500–1000 Hz signals were extracted by digital filtering to present the SKNA (right path). The final output of signal processing is the envelope of SKNA (eSKNA).

The procedure of signal processing is illustrated in Figure 1. The low- and high-frequency components of the raw signal were extracted through digital filtering. The signal obtained after the bandpass filter (1–150 Hz) was designated as ECG, which was further processed by an R-wave peak detector to detect the R–R intervals and to calculate heart rate. On the other hand, the signal obtained after filtering with a bandpass filter (500–1,000 Hz) was SKNA based on our previous work (Kusayama et al., 2020b). The amplitude of SKNA was approximately ±30 μV. To analyze SKNA more instinctively and visually, it was first rectified and then signal-processed by moving average and root mean square (RMS). The parameters for moving average were set as 100-ms window size and 50-Hz moving frequency, i.e., the window advanced every 20 ms. After the first layer processing (moving average), the signal was effectively downsampled to 1/200. The process of moving average can be described with the following equations:


(1) $$\text{MA}=\left[{\text{ma}}_{0},{\text{ma}}_{1},\cdots ,{\text{ma}}_{j}\right]$$

(2) $${\text{ma}}_{a}=\frac{\sum_{i=a}^{m+a-1}{\text{x}}_{i}}{\text{m}}$$

(3) a=0,1,⋯,j

(4) $$\text{j}=\frac{\left(\text{SR}\times \text{T}-\text{m}\right)\times {\text{M}}_{\mathrm{freq}.}}{\text{SR}}$$

Where *j* is the number of samples of MA, *m* is the number of samples in a window, *x*_*i*_ is the *i*th sample value of array X, *SR* is the sampling rate, *M*_*freq*_ is the moving frequency of window, and *T* is the duration of the selected segment in seconds.

The second layer of signal processing was an RMS calculator, which used 100 samples as the window size and moved the windows per sample without downsampling. We defined the final output of signal processing as the envelope of SKNA (eSKNA). The equation of RMS is similar to the moving average. Eqs. (3, 4) still apply.


(5) $$\text{RMS}=\left[{\text{rms}}_{0},{\text{rms}}_{1},\cdots ,{\text{rms}}_{j}\right]$$

(6) $${\text{rms}}_{a}=\sqrt{\frac{\sum_{i=a}^{n+a-1}{\text{ma}}_{i}^{2}}{\text{n}}}$$

Where j is the number of samples of RMS, rms_a_ is the *a*th sample value of RMS, n is the number of samples in a window, and ma_i_ is the *i*th element of moving average array.

The eSKNA was used to label the nerve clusters for further analysis and compared with the heart rate changes. A threshold level was determined similar to the previous study (White et al., 2015). The thresholds were calculated by the following equation:


(7) Threshold=(Baseline-Min)×5+Min

Where *Baseline* is defined as the average of the lower 20 percentile samples in the selected window. *Min* is the minimum value in the selected window.

### Skin Sympathetic Nerve Activity Cluster Definition

**FIGURE 2:**
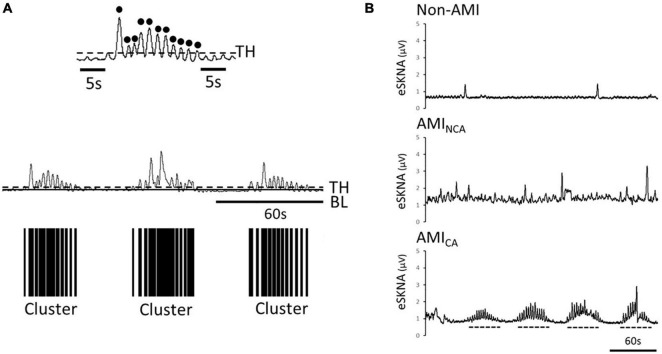
**(A)** Black dots are the defined bursts, which are the peaks above the threshold. Definition of a cluster, which is defined to contain at least five bursts within 10–60 s and without any bursts before/after the cluster in 5 s. The black bars are the clusters found in the eSKNA. The distribution of the black bars shows the clustering phenomenon. TH, threshold; BL, baseline. **(B)** Examples of eSKNA in non-AMI, AMI_NCA_, and AMI_CA_ showing the morphological difference. The non-AMI group is relatively stable compared to the AMI group that shows sporadic bursts. The AMI_NCA_ group has higher variance and irregular bursts. The AMI_CA_ group can observe the obvious clustering phenomenon. Dotted lines indicate the clusters.

The signal amplitude level above the threshold [calculated in eq. (7)] is defined as a single burst. Through the distribution of burst occurrence, we can distinguish whether the bursts were gathered into a cluster, defined by the modified definition of cluster phenomenon of MSNA (Furlan et al., 2000; Marchi et al., 2016). To label the cluster phenomenon objectively, we defined a cluster that contains at least five bursts within 10–60 s and without any bursts before/after the cluster in 5 s, as shown in Figure 2A.

The clusters were considered as the synchronized neural discharge. By labeling the clusters, the AMI subjects were separated into non-cluster appearing (AMI_NCA_) and cluster appearing (AMI_CA_) groups.

### Heart Rate Variability Analysis

The R–R intervals extracted from ECG were used for time- and frequency-domain HRV analyses (Malik et al., 1996). The premature junctional complex, premature ventricular complex, and noise were manually removed to preserve the stable R–R interval sequence (Lippman et al., 1994). The window size of HRV was set as the duration of whole recording. The HRV parameters were calculated using the built-in HRV function of the LabVIEW platform (National Instruments, Austin, TX, United States). The standard setting of HRV calculation followed the suggestions by Rajendra Acharya et al. (2006). The proportion of the number of pairs of successive R–R intervals that differ by more than 50 ms divided by the total number of R–R intervals (pNN50), standard deviation of normal-to-normal beat intervals (SDNN), and the square root of the mean of the squares of the successive differences between adjacent R–R intervals (RMSSD) were used as indices in linear time-domain analysis. The frequency is separated into two sections, including LF (0.04–0.15 Hz) and high frequency (HF, 0.15–0.4 Hz). We use LF/HF ratio, LF power, and HF power as parameters to assess the HRV frequency domain analysis.

### Statistical Analysis

Data were expressed as median, 25th percentile, and 75th percentile for numerical variables and percentage for categorical variables. Prevalence of disease and clinical characteristics were compared between non-AMI and AMI patients by chi-square test. Chi-square tests were also used to examine the association between the SKNA cluster phenomenon and the comorbidities, such as diabetes mellitus, hypertension, dyslipidemia, heart failure, and ST elevation myocardial infarction (STEMI)/non-ST elevation myocardial infarction (NSTEMI) in the AMI group. AMI and non-AMI participants were recruited according to the predetermined sample size, which was estimated based on a two-tailed test with a power of 0.80 and a type I error of 0.05, and an estimated mean difference of 0.40 for SKNA (1.1 for AMI and 0.7 for non-AMI) with a common standard deviation of 0.3. The predetermined samples for AMI and non-AMI participants were set to be equal. The minimal sample size calculated was 40 (20 for each group). The multivariate linear regression model was used to identify the causes of changes in average eSKNA. Correlation between average eSKNA and HRV parameters was analyzed by Spearman test. HRV and average eSKNA were compared between the non-AMI, AMI_NCA_, and AMI_CA_ groups by using Mann–Whitney *U* test. All statistical tests were performed using IBM SPSS Statistics Base version 22 and Stata version 15.

## Results

### Baseline Characteristics of the Subjects

Table 1 shows the baseline characteristics of the study subjects. The prevalence of diabetes, hypertension, and dyslipidemia is higher in the AMI group. The causes of changes in SKNA were discovered by multivariate analysis in Supplementary Table 1, which showed that AMI is a significant factor causing the SKNA to increase. The mean ejection fraction of AMI patients is 57% (Q1–Q3: 49–63%). Five of the 20 AMI patients have the diagnosis of heart failure with reduced ejection fraction when discharged. The max creatine phosphokinase level of AMI patients is 2,578 IU/L (Q1–Q3: 973–4,981 IU/L). Sixty-five percent of AMI patients were ST elevation AMI (STEMI). Thirty-one percent of STEMI patients were inferior wall STEMI. The percentage of the inferior wall STEMI is 33% in the AMI_CA_ group and 36% in the AMI_NCA_ group. The inferior wall STEMI is not correlated with the clustering of SKNA (*p* = 0.888). There is no difference in patient characteristics between the AMINCA and AMICA groups. Typical eSKNA patterns show differences between non-AMI, AMI_NCA_, and AMI_CA_ groups, respectively, in Figure 2B. From the 20 AMI patients, nine patients were identified to have eSKNA burst clusters in the recording. The occurrence of cluster was between 0.01 and 0.03 Hz, and the amplitude of cluster defined by the average of each peak of burst was approximately 3 μV (after RMS processed). On the contrary, eSKNA of the remaining 11 AMI patients showed irregular bursts. The 20 non-AMI subjects only showed sporadic bursts and the level of eSKNA remained stable. There is no association between the SKNA cluster phenomenon and the comorbidities, including diabetes mellitus, hypertension, dyslipidemia, heart failure, and STEMI/NSTEMI in the AMI group (all *p* ≥ 0.178).

### Correlation of Envelope of SKNA and Heart Rate Variability Analysis

**FIGURE 3:**
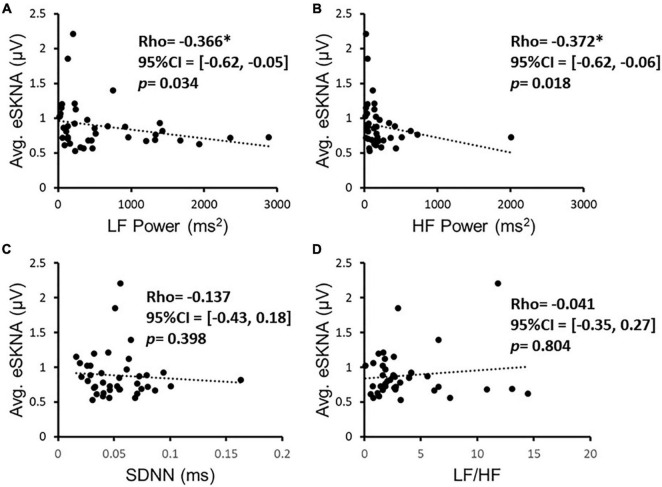
The correlation of eSKNA and the parameters in HRV analysis in 40 study subjects. **(A,B)** Show the significant correlations between eSKNA and LF power, and eSKNA and HF power, respectively. **(C,D)** Show no correlation between eSKNA and SDNN, and eSKNA and LF/HF, respectively. *Statistically significant (*p* < 0.05).

**FIGURE 4:**
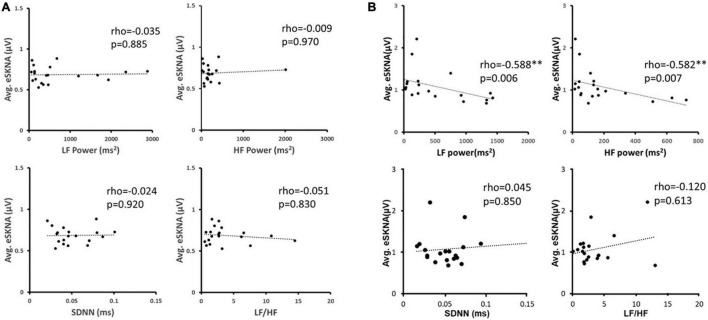
**(A)** The correlation between average eSKNA and HRV parameters in non-AMI groups. **(B)** The correlation between average eSKNA and HRV parameters in AMI groups. ^∗∗^Statistically significant (*p* < 0.01).

We verified the correlation of eSKNA and the parameters in HRV analysis in 40 study subjects. In Figure 3, the correlation between average eSKNA and the parameters in HRV was verified. In Figures 3A,B, the average eSKNA was significantly correlated with LF power (rho = −0.336) and HF power (rho = −0.372) (both *p* ≤ 0.007). However, the average eSKNA showed no significant correlation with SDNN and LF/HF ratio (Figures 3C,D). The average eSKNA was not significantly correlated with pNN50 and RMSSD as well (Supplementary Figure 1). Therefore, we cross-compared the correlation of average eSKNA and the HRV parameters in the non-AMI and the AMI (AMI_CA_ and AMI_NCA_) groups. No HRV parameters were significantly correlated with the average eSKNA in the non-AMI group (Figure 4A). However, the average eSKNA was significantly correlated with LF power and HF power in the AMI group (Figure 4B).

**FIGURE 5:**
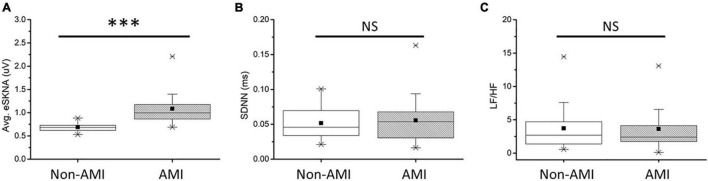
**(A)** Average eSKNA is statistically significant between non-AMI and AMI groups. **(B)** SDNN does not exist statistically significant between non-AMI and AMI groups. **(C)** LF/HF ratio does not exist statistically significant between non-AMI and AMI groups. ^∗∗∗^Statistically significant (*p* < 0.001); NS, not significant.

In Figure 5A, the average eSKNA of non-AMI (median: 0.680; Q1–Q3: 0.617–0.728) was significantly lower than the AMI group (median: 0.997; Q1–Q3: 0.857–1.188). SDNN and LF/HF ratio showed no significant difference in non-AMI and AMI (Figures 5B,C). The pNN50, RMSSD, LF power, and HF power were not significantly different as well (Supplementary Figure 2). Table 2 indicated that the average eSKNA could be an indicator to distinguish between non-AMI and AMI due to eSKNA in non-AMI being significantly lower than either AMI_NCA_ or AMI_CA_ group.

**TABLE 2:** eSKNA and HRV parameters.

	**(1) Non-AMI**		**(2) AMI_NCA_**		**(3) AMI_CA_**		**(1) vs. (2)**	**(2) vs. (3)**	**(1) vs. (3)**
**Parameters HRV (time domain)**	**Median**	**(Q1–Q3)**	**Median**	**(Q1–Q3)**	**Median**	**(Q1–Q3)**	***p*-value**	***p*-value**	***p*-value**
**Linear**									
pNN50 (%)	4.535	(2.262–19.300)	5.991	(0.593–11.111)	3.439	(1.390–18.458)	0.563	0.849	0.706
SDNN (ms)	46	(33–70)	0.056	(26–73)	45	(31–62)	0.710	0.569	0.706
RMSSD (ms)	26.465	(21.868–40.380)	26.310	(13.700–31.21)	25.990	(18.735–44.195)	0.433	0.569	0.925
**HRV (frequency domain)**									
LF power (ms^2^)	382.420	(133.120–1,070.762)	501.466	(46.742–1,329.760)	229.602	(92.829–678.625)	0.741	0.790	0.278
Normalized LF (%)	72.906	(57.447–83.637)	79.834	(64.789–86.776)	65.246	(59.437–70.647)	0.215	0.044*	0.322
HF power (ms^2^)	158.298	(64.490–251.699)	101.671	(17.937–164.380)	135.524	(53.379–360.903)	0.083	0.184	0.777
Normalized HF (%)	27.094	(16.363–42.553)	20.166	(13.224–35.211)	34.754	(29.353–40.563)	0.215	0.044*	0.322
LF/HF	2.691	(1.351–5.451)	3.959	(1.840–6.562)	1.877	(1.483–2.413)	0.215	0.044*	0.322
**eSKNA**									
Average (μV)	0.680	(0.617–0.728)	1.024	(0.852–1.399)	0.972	(0.852–1.161)	<0.001	0.676	<0.001***

*AMI, acute myocardial infarction; AMI_*CA*_, AMI subjects with cluster appearing; AMI_*NCA*_, AMI subjects with non-cluster appearing; eSKNA, envelope of SKNA; HF, high frequency; HRV, heart rate variability; LF, low frequency; LF/HF, the ratio of LF to HF; pNN50, the proportion of the number of pairs of successive R–R intervals that differ by more than 50 ms divided by the total number of R–R intervals; Q1, 25th percentile; Q3 75th percentile; RMSSD, the square root of the mean of the squares of the successive differences between adjacent R–R intervals; SDNN, standard deviation of R–R intervals.*

*Statistically significant (*p* < 0.05). ***Statistically significant (*p* < 0.001).

### Average Envelope of SKNA and Heart Rate Variability Analysis in Non-cluster Appearing and Cluster Appearing Group

**FIGURE 6:**
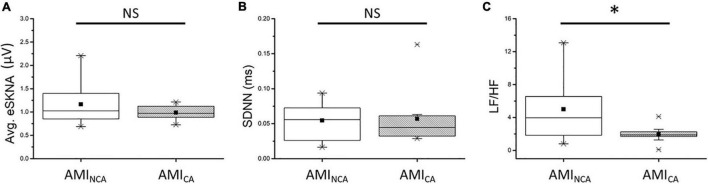
**(A)** No significant difference exists in average eSKNA between AMI_NCA_ and AMI_CA_ (*p* = 0.676). **(B)** No significant difference exists in SDNN between AMI_NCA_ and AMI_CA_ (*p* = 0.569). **(C)** The LF/HF ratio is statistically significant between AMI_NCA_ and AMI_CA_ (*p* = 0.044). ^∗^Statistically significant (*p* < 0.05); NS, not significant.

The eSKNA was used as the average nerve discharge amplitude analysis for the assessment of non-invasive SKNA. In Figures 6A,B, the average eSKNA and SDNN showed no significant difference between AMI_NCA_ and AMI_CA_ groups. In Figure 6C, the LF/HF ratio of AMI_NCA_ shows significantly higher value and variability (median: 3.959; Q1–Q3: 1.840–6.562) compared with the AMI_CA_ group (median: 1.877; Q1–Q3: 1.483–2.413). Table 2 provides detailed HRV parameters and eSKNA statistical data, including median, 25th percentile, and 75th percentile. Normalized LF, normalized HF, and LF/HF ratio showed statistical significance between AMI_NCA_ and AMI_CA_. The difference between groups cannot be found in HRV time-domain parameters and the power in the frequency domain.

## Discussion

We hypothesized the higher ANS regulation ability in AMI patients could cause synchronized neural discharge in non-invasive SKNA. According to our definition of bursts and clusters, we observed the cluster phenomenon of eSKNA in 9 of 20 AMI patients in 5-min baseline recordings and no eSKNA clusters were observed in the non-AMI group. AMI_CA_ group has significantly lower normalized LF (median: 65.246; Q1–Q3: 59.437–70.647) and LF/HF ratio (median: 1.877; Q1–Q3: 1.483–2.413) than AMI_NCA_ group (median: 79.834; Q1–Q3: 64.789–86.776 and median: 3.959; Q1–Q3: 1.840–6.562, respectively), suggesting that the eSKNA clustering may indicate the lower sympathetic tone in the AMI patients, which might further reflect the regulation of ANS in AMI. The AMI_CA_ group also has significantly higher normalized HF (median: 34.754; Q1–Q3: 29.353–40.563) than the AMI_NCA_ group (median: 20.166; Q1–Q3: 13.224–35.211).

Kasama et al. (2007) and Han et al. (2012) also reported that, after MI, there was left ventricular and left stellate ganglia (SG) electroanatomic remodeling, which could account for the correlation between SKNA, cardiac and ANS remodeling, and arrhythmias. We previously demonstrated that SKNA is able to estimate the SG nerve activity in canines (Jiang et al., 2015). Recently, Kusayama et al. (2019) reported the temporal correlation between cardiac arrhythmias and SKNA. It revealed that the frequent arrhythmias were associated with large and sustained SKNA. Combining the evidence above, we believed that it is justified to use SKNA as an index of sympathetic tone in cardiovascular research. The clustering phenomenon found in our study supports the interpretations in other studies (Kasama et al., 2007; Han et al., 2012; Kusayama et al., 2019) that sympathetic nerves would coordinate and synchronize when the ANS balance was altered. The clustering of MSNA has been observed at lower body negative pressure and during the orthostatic stimulus (Furlan et al., 2000; Marchi et al., 2016; Ang and Marina, 2020). However, the distribution of nerve discharge has never been mentioned in non-invasive SKNA studies. Our study is the first to show the clustering phenomenon in non-invasive SKNA recording. Contrary to the previous MSNA study, which showed the cluster phenomenon developed under increased sympathetic tone, the cluster phenomenon in our study did not correlate with higher SKNA. Indeed, there is no SKNA difference but only lower normalized LF and LF/HF ratio and higher normalized HF in AMI_CA_ than in the AMI_NCA_ group. The higher normalized HF and lower normalized LF and LF/HF ratio in AMI_CA_ might reflect autonomic regulation when patients face stressed conditions such as AMI.

We examined the correlation of average eSKNA and HRV parameters to verify eSKNA and found that it is a valid surrogate marker to assess ANS status in AMI patients. In Figure 4B, we confirm that the contribution of statistically significant correlation of average eSKNA is from the AMI group, including average eSKNA to pNN50, RMSSD, LF power, and HF power, which are the parasympathetic activity-related indices (Malik et al., 1996). Unlike the positive correlation between LF components and MSNA in normal humans (Pagani et al., 1997), the non-AMI group does not show any significant correlation between eSKNA and HRV parameters (Figure 4A). After the cross-comparison, eSKNA reveals a higher correlation to HRV in AMI than the non-AMI group. According to HRV description in the research of Rajendra Acharya et al. (2006), our results support the notion that eSKNA is a credible indicator to assess ANS in AMI patients.

In non-invasive SKNA, MSNA, and skin sympathetic nerve activity recorded by microneurography (SSNA) studies, the comparison between subjects was usually based on average nerve activity, integrated nerve activity, and the bursts per minute. Swierblewska et al. (2010) proposed that the subjects with excessive pulse wave velocity had significantly greater MSNA than subjects with regular pulse wave velocity. Their definition of MSNA has used the bursts per minute as a quantitative indicator. A previous study showed a right lateralization of sympathetic activity from the vessels accessed by the bilaterally MSNA recording (Diedrich et al., 2009). The method we used to measure SKNA showed the same trend with the right leg SSNA in response to the sympathetic stimulations (Kusayama et al., 2020b). In the study of patients with atrial arrhythmias by Uradu et al. (2017), the average SKNA revealed the increase during atrial fibrillation and atrial tachycardia episodes’ onset and termination. Kusayama et al. (2019) concluded that the large and sustained SKNA were associated with frequently ventricular tachycardia and ventricular fibrillation. The parameters, such as average nerve activity, integrated nerve activity, and the bursts per minute, provide a macroscopic description of SKNA. In Figure 6A, the aim of analyzing the average SKNA between AMI_NCA_ and AMI_CA_ groups was to examine whether the cluster-caused physiological variations could also be observed in the amplitude of average nerve activity or not. The average eSKNA showed no significant difference between AMI_NCA_ and AMI_CA_ groups. The statistical analysis showed that average SKNA can broadly distinguish between non-AMI and AMI but cannot identify the subtler variations in ANS such as clustering phenomenon in the AMI patients. Along with the studies mentioned above, SKNA was correlated with AMI and cardiac arrhythmia; thus, we believe that SKNA is able to represent cardiac sympathetic activity. Furthermore, the results demonstrated that the SKNA clustering is a unique temporal pattern of ANS synchronized discharge. The temporal clustering pattern provides an additional indicator for non-invasive SKNA-related studies. As shown in a previous study (Furlan et al., 2019), the loss of MSNA clustering preceding the vasovagal syncope might indicate that clustering changing from presence to absence is related to a pathophysiological state. Along with our study, the absence of SKNA clustering is associated with higher LF/HF ratio than the presence of SKNA clustering, which might indicate that the absence of SKNA clustering is related to a pathophysiological state. However, we need more follow-up data in the future study to consolidate the role of SKNA clustering in AMI.

Due to operational limitations, the two processes of moving average and RMS in signal processing could cause a slight phase delay. However, the delay time is minor compared to the clustering phenomenon (approximately 3–5 min). A potential limitation is that our study cannot directly establish a causal relationship between clustering phenomenon and arrhythmia events. Second, our study is limited to men. SKNA patterns may be different in women. Third, the data did not include the patients after surgery in 1 year. According to our research, the cluster occurrence and amplitude may change during AMI recovery process. In future studies, we will continuously collect the data every month to deduce SKNA morphological changes’ implications. Fourth, the *N* value is not large enough to depict complete statistic results. In our analysis, some of the *p*-values were close to 0.05 level of significance. We want to demonstrate the phenomenon of eSKNA clustering as an important biomarker of ANS regulation. More studies are required to establish its clinical relevance. Fifth, the signals recorded by the neuECG might be the mixes of electrical activities from the motor, sensory and autonomic nerves. However, the subjects were rested in a quiet space and asked to stay calm during recording. The motion artifacts and sensory electrical activities were minimized and should not affect the study result since both AMI and non-AMI groups were recorded in the same condition. Sixth, the present study did not include clinical interventions. Further interventions such as orthostatic or pharmacological challenges should be considered in future studies. Seventh, the correlation between SKNA and HRV indexes should be carefully addressed since only the short-term HRV was analyzed. Extended studies with longer follow-up periods are needed to clarify the SKNA/HRV correlation and the physiological significance of temporal clustering in AMI patients. Eighth, since there was a large portion of patients in the AMI group with comorbidities, such as diabetes mellitus (50%), hypertension (55%), and dyslipidemia (80%), the presence of SKNA cluster phenomenon in the AMI group might partially be explained by these comorbidities. However, when we checked the association between the SKNA cluster and these comorbidities in the AMI group, no association was found. Thus, the comorbidities might have limited effects on SKNA clustering. Finally, the mechanism of the cluster phenomenon in SKNA in AMI patients remains to be determined.

## Conclusion

This is the first study to identify SKNA clustering phenomenon in AMI patients. Such a synchronized nerve discharge pattern could be detected with the non-invasive recording of SKNA signals. SKNA clustering phenomenon could represent the specific changes in ANS regulation and ANS homeostasis in AMI patients. SKNA temporal clustering could be a novel biomarker to classify ANS regulation ability in AMI patients.

## Data Availability Statement

The original contributions presented in the study are included in the article/Supplementary Material, further inquiries can be directed to the corresponding author/s.

## Ethics Statement

The studies involving human participants were reviewed and approved by the Institutional Review Board of Kaohsiung Medical University Hospital. The patients/participants provided their written informed consent to participate in this study.

## Author Contributions

W-CT and S-FL contributed to the conception and design of the study. C-HL performed the statistical analysis. CL wrote the first draft of the manuscript. CL, W-CT, and S-FL wrote the sections of the manuscript. All authors contributed to manuscript revision, read, and approved the submitted version.

## Conflict of Interest

The authors declare that the research was conducted in the absence of any commercial or financial relationships that could be construed as a potential conflict of interest.

## Publisher’s Note

All claims expressed in this article are solely those of the authors and do not necessarily represent those of their affiliated organizations, or those of the publisher, the editors and the reviewers. Any product that may be evaluated in this article, or claim that may be made by its manufacturer, is not guaranteed or endorsed by the publisher.
