# Supplementary material for: Temporal Clustering of Skin Sympathetic Nerve Activity Bursts in Acute Myocardial Infarction Patients
Source: Front Neurosci. 2021 Nov 30;15:720827. doi: 10.3389/fnins.2021.720827 (PMC8669957; doi:10.3389/fnins.2021.720827)
Supplement: Supplementary file 1 [file Data_Sheet_1.docx]

Supplementary Material


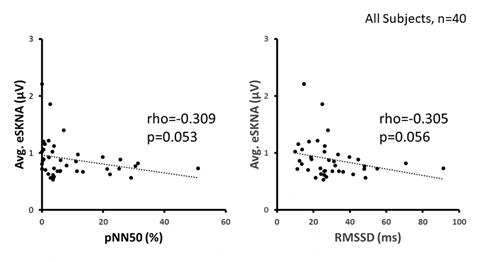


Figure S1. No significant correlation exists between either average eSKNA and pNN50 or average eSKNA and RMSSD in all subjects.


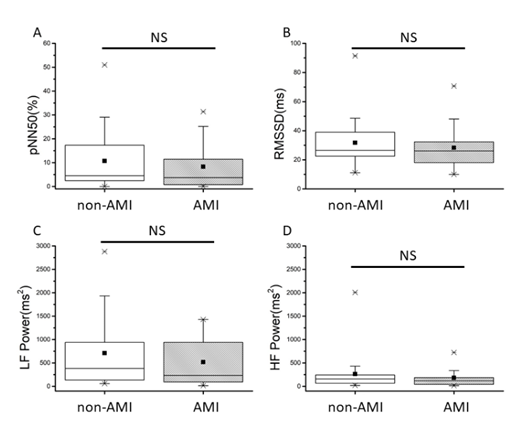


Figure S2. No significant difference exists in pNN50, RMSSD, LF power, and HF power between non-AMI and AMI groups. NS, Not Significant.

| **Supplemental table 1.** **The adjusted mean (adj. β) of skin sympathetic nerve activity (SKNA) associated with demographic, lifestyle, and clinical factors** | | | | | | | |  |
| --- | --- | --- | --- | --- | --- | --- | --- | --- |
| **Characteristics** | **Model 1^a^ (0.1μV)** | | **P value** |  | **Model 2^b^ (0.1μV)** | | **P value** | |
|  | **adj. β** | **95% CI** |  |  | **adj. β** | **95% CI** |  |  |
| **Demographic and lifestyle factors** |  |  |  |  |  |  |  | |
| Age (mean± SD), year | 0.057 | (-0.023, 0.137) | 0.154 |  | 0.056 | (-0.02, 0.131) | 0.145 | |
| Cigarette smoking, yes vs. no | 0.991 | (-1.367, 3.349) | 0.399 |  | -0.967 | (-3.307, 1,374) | 0.406 | |
| Alcohol drinking, yes vs. no | 0.285 | (-2.277, 2.847) | 0.822 |  | 0.215 | (-1.984, 2.413) | 0.843 | |
| Betel-quid chewing, yes vs. no | 0.535 | (-2.650, 3.721) | 0.735 |  | 1.302 | (-1.428, 4.031) | 0.338 | |
| BMI, kg/m^2^ | 0.227 | (-0.073, 0.527) | 0.133 |  | -0.004 | (-0.299, 0.292) | 0.980 | |
| **Clinical factors** |  |  |  |  |  |  |  | |
| Hypertension, yes vs. no |  |  |  |  | -2.413 | (-4.703, -0.123) | 0.04 | |
| Diabetes mellitus, yes vs. no |  |  |  |  | 0.545 | (-2.405, 3.495) | 0.709 | |
| Dyslipidemia, yes vs. no |  |  |  |  | 1.704 | (-0.558, 3.966) | 0.134 | |
| AMI, yes vs. no |  |  |  |  | 4.366 | (1.504, 7.228) | 0.004 | |
| AMI, acute myocardial infarction; BMI, body mass index; CI, confident interval; SD; standard deviation.  ^a^Model was obtained from multiple linear regression models adjusted for age, gender, cigarette smoking, alcohol drinking, betel-quid chewing and BMI.  ^b^Model was obtained from Model 1 and additionally adjusted for adjusted for hypertension, diabetes mellitus, dyslipidemia and AMI. | | | | | | | |  |
